# A blended group intervention to promote social connectedness and wellbeing among international university students: an exploratory study

**DOI:** 10.3389/fpsyg.2024.1497544

**Published:** 2024-11-27

**Authors:** Sabrina Cipolletta, Ilaria Tedoldi, Silvia Caterina Maria Tomaino

**Affiliations:** Department of General Psychology, University of Padua, Padua, Italy

**Keywords:** blended intervention, loneliness, social isolation, university students, young adults, wellbeing

## Abstract

**Introduction:**

Loneliness is a prevalent issue among international university students, often exacerbated by cultural and linguistic barriers. This pilot study aims to assess the feasibility, acceptability and impact of a blended intervention to promote international students’ social connectedness and well-being.

**Methods:**

A sample of 49 international students from the University of Padua (Italy) was recruited. The study followed the methodology of a non-randomized controlled trial comparing a blended intervention (comprising group activities and online self-help materials) with two other active conditions (self-help only and peer-to-peer interventions) and a control condition at two times (baseline and at 8 weeks). Participants completed a survey to assess their satisfaction with the interventions, changes in their interactions and wellbeing. They also filled in some questionnaires to measure anxiety, depression, perceived social support, loneliness and satisfaction with life. A mixed-method analysis was conducted.

**Results:**

Results showed that interventions involving in-person activities had significant advantages over self-help intervention in terms of interaction improvement and a higher number of relationships. Participants perceived self-help materials as more relevant, satisfactory, and functional within the blended group compared to the self-help group. Contrary to the control group, the blended and peer-to-peer groups reported lower scores on the standardized measures of loneliness, anxiety, and depression, and higher scores on satisfaction with life, collected pre- and post-intervention. The thematic analysis of the answers to the open-ended questions showed that in-person group activities provided the opportunity to compare themselves with peers and have a direct experience of new social connections.

**Discussion:**

The findings highlight the importance of translating insights from self-help materials into active and direct social experiences, to reduce loneliness through the emergence of new perspectives and shared meaning making.

## 1 Introduction

International students are individuals who leave their home country to pursue higher education abroad, seeking enhanced academic opportunities and expanding their career prospects (Güneş and Aydar, 2019). Recent data indicates a significant rise in internationally-mobile students, with over 6.3 million recorded globally in 2020 compared to 2 million in 2000 (UNESCO Institute for Statistics, United Nations Children's Fund, and The World Bank, 2022), marking a 70% increase over the past decade (OECD, 2022). While studying abroad offers enriching learning experiences (Bilecen et al., 2023), it also poses challenges in adapting to new educational systems and socio-cultural environments. This process, known as acculturation (Wahyuningtyas et al., 2021), can induce stress, with adverse implications for psychological and social well-being (Alshammari et al., 2023; Bender et al., 2019; Koo et al., 2021; Pang, 2020; Serrano-Sánchez et al., 2021). Acculturative stress may lead to the development of depression and anxiety symptoms (Alshammari et al., 2023; Brunsting et al., 2018; Jung et al., 2007; Koo et al., 2021; Newton et al., 2021; Pang, 2020), and this evidence is supported by numerous studies indicating that, compared to other student groups, international students exhibit a higher incidence of mental health issues (Alharbi and Smith, 2018; Koo et al., 2021; Newton et al., 2021). Additionally, the literature underscores that loneliness is one of the primary challenges arising from poor adjustment to new environments, largely due to cultural disparities and language barriers, and that international students are proved to be particularly susceptible to social isolation and loneliness (Alharbi and Smith, 2018; Bilecen et al., 2023; Serrano-Sánchez et al., 2021; Wawera and Mccamley, 2020), often exacerbated by discriminatory treatment (Alshammari et al., 2023; Sun et al., 2020).

The distressing experience of loneliness encompasses a strong sense of social pain, dysphoria, emptiness, and worthlessness (Cacioppo et al., 2014; Matthews et al., 2016), stemming from a perceived deficiency in relationships (with individuals, groups, or communities) mainly related to the qualitative aspects of their social networks (Matthews et al., 2019; Perlman and Peplau, 1981; Wigfield et al., 2022). Chronic loneliness has been associated with the development and exacerbation of depressive symptoms (Cacioppo et al., 2014; Pitman et al., 2018), anxiety, alcohol abuse, cognitive decline, dementia in older age, and an increased mortality risk (Christiansen et al., 2021; Hawkley and Cacioppo, 2010; Matthews et al., 2016; Rico-Uribe et al., 2018). Long-term studies also link loneliness to a higher risk of physical illnesses such as cardiovascular disease, hypertension, asthma, migraine, tinnitus, osteoarthritis, and slipped disk (Christiansen et al., 2021; Hawkley and Cacioppo, 2010; Matthews et al., 2019). Considering the extensive evidence, loneliness emerges as a significant public health concern, especially when considering the long-term consequences of the recent COVID-19 pandemic (Lampraki et al., 2022). Pandemic-related social restrictions contributed to increased levels of loneliness, depression, anxiety, insomnia, substance use, and suicidal attempts among young adults in general (Glowacz and Schmits, 2020; Hawes et al., 2022; Sewall et al., 2022) and college students in particular (Buizza et al., 2022; Carvalho et al., 2022; Elharake et al., 2023; Haikalis et al., 2022). Lockdowns and border closures particularly heightened the vulnerability of international students, intensifying feelings of loneliness, social isolation, and exacerbating their negative impact on well-being (Alaklabi et al., 2021; Bilecen, 2020; Zhao et al., 2022). It’s evident that urgent attention must be paid and support must be provided in order to address this distressing trend among international students. Psychological and social interventions are imperative to ensure the well-being and mental health of these students, particularly in light of the lasting effects of the recent pandemic.

Loneliness, while not an inherent trait, is shaped by life experiences, and studies showed the effectiveness of psychological and psychosocial interventions in mitigating loneliness (Bessaha et al., 2020). However, understanding the factors determining their benefits is challenging due to diverse intervention designs, theoretical foundations, delivery modalities, focal points, and the strategies employed (Eccles and Qualter, 2021; Hickin et al., 2021; Masi et al., 2011; Osborn et al., 2021; Zagic et al., 2022). Despite controversies, the current literature suggests that when compared to individual sessions, group interventions can be more successful in improving the quality of social connections by providing the opportunity to increase the number of social contacts through direct in-person experiences (Eccles and Qualter, 2021; Masi et al., 2011; Zagic et al., 2022). They provide opportunities to access sources of reciprocal social support and motivate lonely people to promote their own well-being while mediating stress responses (Bessaha et al., 2020; Zagic et al., 2022). Social support, defined as the assistance, protection and supportive behavior provided to others (i.e., individuals, groups, and the broader community) through social ties (Brunsting et al., 2021; Langford et al., 1997; Ozbay et al., 2007; Wilson et al., 2020), stands as significant protective factors in overcoming acculturative stress, playing a crucial role in the acculturation process (Bender et al., 2019; Billedo et al., 2019; Cipolletta et al., 2022; Hofhuis et al., 2019; Nakao, 2019; Pang, 2020). According to the literature, a combination of psychoeducation, counseling, and social support intervention in group delivery modality appears to be the most suitable approach for supporting lonely international students (Carr et al., 2003; Dipeolu et al., 2007). Group interventions targeting this specific group can provide a sense of belonging and shared social identification (Bessaha et al., 2020; Gold et al., 2019; Haslam et al., 2019; Masi et al., 2011; Zagic et al., 2022) by normalizing common experiences and alleviating experiences of isolation (Carr et al., 2003; Yakunina et al., 2011), although still dealing with different disadvantages such as problems with the group composition and engagement, cultural and linguistic differences leading to conflicts and misunderstandings and low participation potentially connected to high levels of social anxiety or loneliness. There are also some examples of online interventions for university (Charbonnier et al., 2022) and international college students (Balci et al., 2023) showing significant improvements in overall well-being and anxiety, while at the same time pointing out potential limitations such as high drop-out rate (50%), low adherence (only 40% completed the intervention), unexplored long-term effects, and the need for further explorations. As digital and group intervention -if separately implemented- show different disadvantages, blended interventions (combining online and face-to-face modalities) could represent an innovative and resourceful opportunity to respond to international students’ needs and challenges, facilitating help-seeking behaviors and adherence, and reducing barriers to mental healthcare (De Witte et al., 2021; Evans-Lacko et al., 2022), thus combining the advantages of online and face-to-face modalities while minimizing their disadvantages (Erbe et al., 2017).

This pilot study is the first to have tested a blended intervention, comprising online self-help activities and face-to-face group sessions to improve well-being and reduce the increasing experiences of loneliness and social isolation among international students. Given the inherent complexity in both the experience of loneliness and the situation international students face, a blended intervention is proposed, consisting of two different components: participation in face-to-face group activities conducted by a professional, and the use of online self-help materials. The theoretical framework with regard to group activities and self-help materials which has been developed is that of Personal Construct Psychology (PCP) (Kelly, 1955). This framework advances a perspective that centers on the subjective construction of wellbeing rather than a pre-defined and normative notion of wellbeing. The proposed activities aim to explore personal beliefs and identify the personal constructs that can promote wellbeing and social connections, while experiencing new ways of interacting with others within a group. To comprehensively evaluate the effects of these activities, the study introduced and compared two other active conditions: the self-help intervention involving access solely to self-help materials, and peer-to-peer intervention, involving participation in group activities facilitated by peers, plus an inactive control group.

The study aims to evaluate the impact of the blended intervention on loneliness, social support and well-being among international students at an Italian university. Additionally, it seeks to investigate which intervention modality (blended, face-to-face or online) and characteristics might be most effective within this population. Lastly, given its pilot nature, the study assesses the acceptability and relevance of the proposed interventions. In line with the literature presented above, the authors hypothesize that the blended intervention will be evaluated as more useful and accessible than the other kinds of interventions, and that the blended and peer-to-peer interventions will be more effective than the online and no intervention approach in promoting participants’ social connectedness and wellbeing.

## 2 Materials and methods

The present study followed the methodology of a randomized controlled trial (RCT). However, due to the small sample size and difficulties recruiting participants, participant randomization was not possible, leading to an acknowledgement of the potential weaknesses inherently present in non-randomized study for psychological research. The study is therefore a non-randomized pilot study reporting a group comparison.

The present research project was registered on ClinicalTrial (Protocol N. NCT05867758) and was approved by the Ethical Committee for the Psychological Research of the University of Padova (Protocol N. 5112).

### 2.1 Participants

An initial (registered at baseline, T0) total number of 49 participants took part in the present study, showing a mean age at the baseline (T0) of 25.10 (SD = 3.44) years. The majority are female (*N* = 29, 59.18%) and participants are of different nationalities (primarily from non-European countries). With regard to their degree programs, participants are enrolled in a bachelor’s (*N* = 14, 28.57%) or single cycle degree (*N* = 1, 2.04%), in a master’s degree (*N* = 24, 48.98%) or in a PhD international program (*N* = 10, 20.41%). Of the initial sample, 27 participants (55.10%) did not complete the intervention, with a rate of 44.44% (*N* = 4) for the blended group and 53.85% (*N* = 7) for the self-help group. In the peer-to-peer group, the rate was 28.57% (*N* = 2), while the control group had a rate of 70%. As reported by the participants, the motivation to not complete the activities were different, such as: geographical impediments for in-presence activities (blended and peer-to-peer), activities overlapping with academic lessons and/or exam session (blended and peer-to-peer), expectations regarding the activities not being met (e.g., the expectation that the participation would have provided academic credits or that participation might have been occasional). Non-completer’s data have been excluded from the analysis.

The sample of treatment completers (registered at the end of the intervention, T1) was composed of 22 participants with a global mean age of 24.82 (SD = 3.17) years. Of these, 45.45% are male (*N* = 10) and 54.55% female (*N* = 12). Participant characteristics of the four groups are reported in Table 1.

**Table 1:** Treatment completers’ characteristics.

Characteristic		Blended group (*N* = 5)		Self-help group (*N* = 6)		Peer to Peer group (*N* = 5)		Control group (*N* = 6)		Total (*N* = 22)	
		*N*	%	*N*	%	*N*	%	*N*	%	*N*	%
Gender	Male	1	20.00	3	50.00	4	80.00	2	33.33	10	45.45
	Female	4	80.00	3	50.00	1	20.00	4	66.67	12	54.55
Nationality	European	0	0.00	0	0.00	0	0.00	2	33.33	2	9.09
	Non-EU	5	100.00	6	100.00	5	100.00	4	66.66	20	90.90
Degree program	Bachelor	2	40.00	0	0.00	1	20.00	3	50.00	6	27.27
	Master	3	60.00	1	16.67	4	40.00	3	50.00	11	50.00
	PhD	0	0.00	5	83.33	0	0.00	0	0.00	5	22.73
Mean age		22.60 (SD = 1.95)		28.17 (SD = 2.99)		24.80 (SD = 2.49)		23.33 (SD = 2.07)		24.82 (SD = 3.17)	
Age range		20–25		23–31		23–29		20–26		20–31	

The inclusion criteria for participants were: being an international student regularly enrolled in a degree or a PhD program of the University of Padua, being over 18 years of age, living in Padua or province during the research period, and fluent in speaking and understanding English. A written informed consent was obtained from all the participants included in the study.

Participants were recruited by posting notices on university bulletin boards and involving university services dealing with international students (Global Engagement Office, the Inclusion Office, and the Tutor Buddies service), which allowed the intervention to be promoted on WhatsApp groups by contacting students’ representatives of international degree programs. The international students involved were asked about their willingness to participate in the interventions proposed and, based on their availability and preferences, they were assigned to the various conditions.

In total, 64 international students provided their email addresses or contacted the research team to express their interest in participating in the scheduled intervention. Those who did not confirm their interest in participating in the interventions after being contacted via email were assigned to the control condition.

### 2.2 The intervention conditions

**Figure 1:**
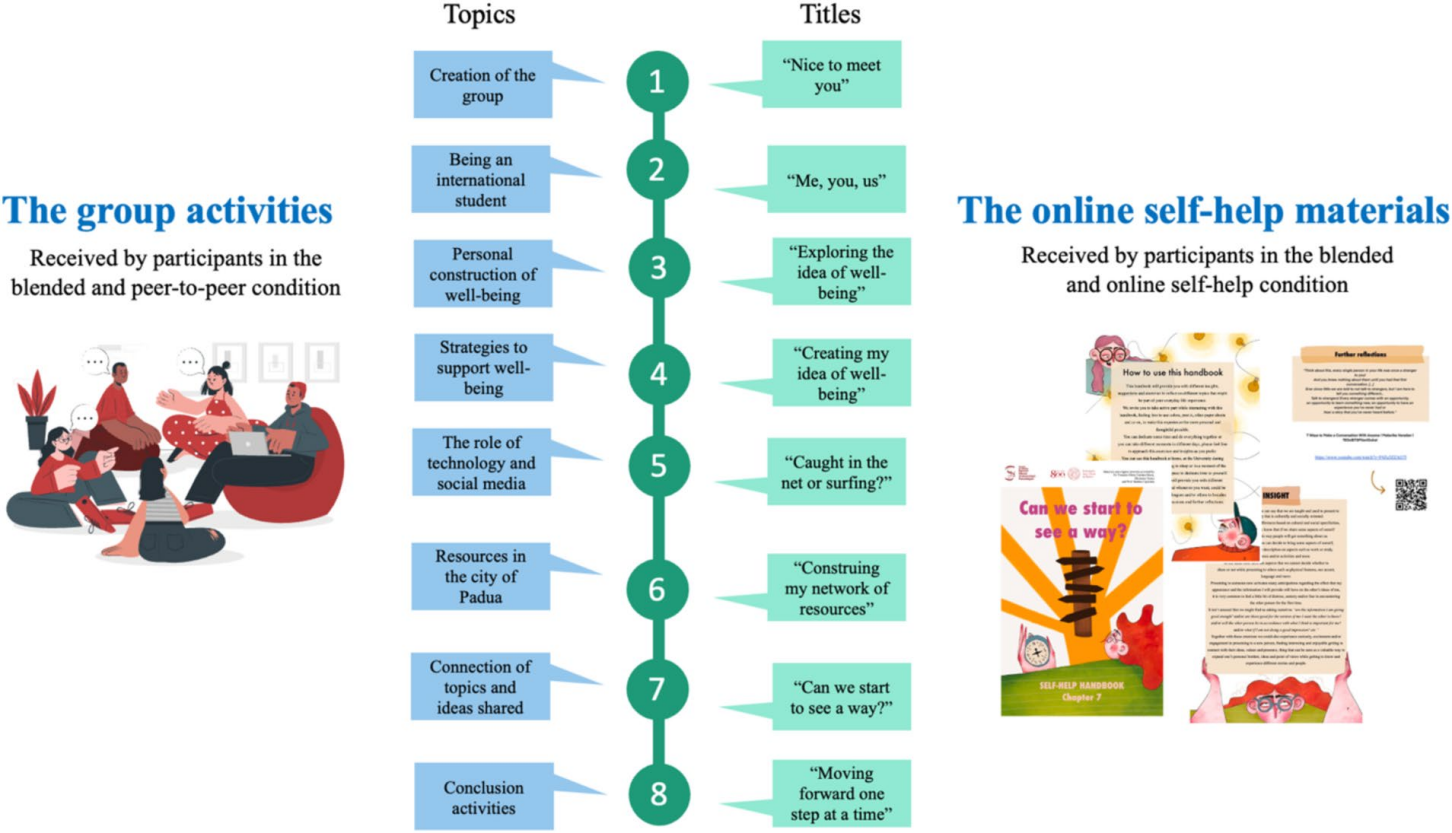
The contents of group sessions and the titles of the chapters of the online material.

The interventions encompassed three active conditions (blended, online self-help and peer-to-peer) including two distinct activities: in-person group sessions and online self-help materials. The blended intervention provided participants with both activities, whereas the self-help intervention provided online materials only, with no in-person sessions. The peer-to-peer intervention, on the other hand, was limited to in-person group activities. The structure of the planned activities remained consistent among groups (see Figure 1).

The blended condition comprised both in-presence activities and online materials lasting 8 weeks in total: participants took part in 8 in-presence group sessions and received in parallel 8 weekly chapters of the self-help materials. During the in-presence group sessions participants were invited to reflect and discuss together a range of different topics such as their experience as international students, personal meanings of wellbeing and activities to support it, modalities to create meaningful relationships with peers while living abroad, resources to overcome practical and personal difficulties while abroad, etc. Each session lasted around 90 min and was facilitated by a psychologist (S.C.M.T.) and a trainee psychologist. The activities were supervised by a psychotherapist (S.C.). At the end of each session, the group was invited to create together an artwork aimed at elaborating a group perspective on the specific topic of the day. This activity was meant to support the elaboration and creation of a common perspective, fostering collaboration and connection between the group members.

The self-help condition comprised 8 chapters of self-help materials, each of which was emailed to participants on a weekly basis. The materials comprised activities, games, texts and digital resources (e.g., TEDx, YouTube videos) regarding different topics such as social networking with peers, creating and maintaining valuable relationships, construing resources and modalities to support one’s wellbeing, construing one’s network of local resources while living abroad, etc. and were meant to support international students’ reflections with regard to different topics. Many of the activities proposed invited the reader to involve peers, friends, family, etc., supporting an elaboration of contents not only in solitude, but also with others.

The peer-to-peer condition comprised 8 in-presence group sessions lasting around 90 min. The structure and contents of the group activities were the same as of those the blended group condition, but without the online self-help materials. The difference was in the role of the facilitators of the group, as the peer-to-peer condition was facilitated by two trainee psychologists (I.T. and another colleague) that at the time of the intervention were still university students. The activities were supervised by a psychotherapist (S.C.) and a psychologist (S.C.M.T.).

Finally, the control condition was a non-active condition which served as a reference, and which was expected to be stable over time. It was used for a more comprehensive evaluation of the interventions and did not involve any specific activity or intervention. The intervention was carried out between November 2022 and May 2023.

### 2.3 Measures

Data were collected using five distinct self-report questionnaires and a final survey. Three questionnaires assessed wellbeing (Satisfaction with Life Survey, SWLS, Diener et al., 1985), loneliness (Ucla Loneliness Scale-6, ULS-6, Neto, 2014), and perceived social support (Multidimensional Scale for Perceived Social Support, MSPSS, Zimet et al., 1988). Two self-report questionnaires assessed anxiety and depressive symptoms (General Anxiety Disorder-7, GAD-7, Spitzer et al., 2006 and Patient Health Questionnaire-9, PHQ-9, Kroenke et al., 2001). Data were collected anonymously at the start (T0) and the end of the intervention (T1) and were matched using an identification code created *ad hoc* by participants during the study. Depending on the group condition, participants completed the questionnaires in paper format (blended and peer-to-peer) or online (self-help and control group) using the Qualtrics platform.

At the end of the intervention, the participants in the three active condition groups completed a final evaluation survey. This was created *ad hoc* to evaluate the satisfaction and relevance of the intervention provided, and was administered online using the Qualtrics platform. The evaluation survey was composed of a total of 47–57 items (depending on the group condition), collecting data about living conditions, the use of psychological assistance, satisfaction and relevance of the intervention, subjective improvement in well-being and in the use of coping resources, the frequency of activities performed individually or with others (leisure activities, study activities, or sports), and others. The survey also comprised six open questions exploring motivations in terms of participation, strengths and weaknesses of the activities, the most enjoyed activities, suggestions to improve the intervention, and the final takeaway of the experience. Personal opinions, subjective evaluations, and feedback from the participants provided insights for potential future modifications and further exploration.

### 2.4 Data analysis

Given the small number of participants, the researchers used non-parametric tests for the comparisons between and within the groups and the effect size to understand how important the observed differences were. A Kruskal-Wallis analysis was conducted to assess the presence of differences between groups on the questionnaire to assess participants’ satisfaction with the intervention and their evaluation of its effectiveness. Wilcoxon test was used to assess the presence of differences between baseline (T0) and post-intervention stages (T1) for each condition on the standardized questionnaires. Given the high non-completers’ rate, an additional U-Mann Whitney test assessed for any significant differences between the final sample and the non-completers’ sample.

The *p* value was set at 0.05 and the effect size was calculated using Spearman correlation for Wilcoxon and U-Mann Whitney test and epsilon squared (ε^2^) for Kruskal-Wallis analysis. Quantitative analyses were all performed using Jamovi software version 2.3.24.

Data derived from the open-ended questions were qualitatively analyzed using a thematic analysis approach (Braun and Clarke, 2006), a flexible method which allows the user to describe a data set, summarizing key features, and reporting thematic patterns within data. Two coders identified the themes present in the answers to the questions and grouped them in thematic areas. This approach was used to evaluate the participants’ opinions with regard to the interventions, and to interpret any differences in satisfaction and relevance between groups.

## 3 Results

### 3.1 Satisfaction with the intervention and self-evaluation of its effects

The mean scores on the final survey show an overall higher level of satisfaction with the intervention in the blended and peer-to-peer groups compared to the self-help group, but the differences were not statistically significant. As reported in Table 2, the satisfaction with the group activities did not vary significantly between the blended and peer-to peer groups, as was the case with the relevance of the group content and the functionality of group organization. However, a significant difference was found in the outcomes of expectation fulfillment, indicating that the expectations regarding the intervention were more fulfilled in the blended group. Moreover, the blended group reported significantly higher satisfaction compared to the self-help group regarding the subjective relevance of self-help materials. Significant differences between groups also arose in terms of the subjective relevance of self-help materials content and in the functionality of the self-help materials. All these comparisons, even if they were not significant, showed a large effect size (ε^2^ ≥ 0.26), thus suggesting an effect of the modality of the intervention on these measures, except for the satisfaction with the group activities that showed a small effect of the modality (blended or peer-to-peer).

**Table 2:** Group comparison in satisfaction scores collected in the final survey on a 7-point scale.

	Group	*N*	*M* (SD)	χ^2^	*p*-value	ε^2^
Total satisfaction	Blended	5	6.40 (0.55)			
	Peer-to-peer	5	6.20 (1.30)	3.21	0.20	0.27
	Self-help	3	5.33 (0.76)			
Expectation	Blended	5	6.80 (0.45)			
	Peer-to-peer	5	5.80 (1.30)	6.25	0.04	0.52
	Self-help	3	4.50 (0.50)			
Group activity satisfaction	Blended	5	6.60 (0.55)	0.06	0.81	0.006
	Peer-to-peer	5	6.40 (0.89)			
Relevance of group content	Blended	5	6.60 (0.55)	1.04	0.31	0.12
	Peer-to-peer	5	6.00 (1.00)			
Functionality of group organization	Blended	5	6.60 (0.55)	1.56	0.21	0.17
	Peer-to-peer	5	5.80 (1.10)			
Self-help materials satisfaction	Blended	5	6.80 (0.45)	5.75	0.002	0.82
	Self-help	3	4.83 (0.29)			
Relevance of self-help materials’ contents	Blended	5	6.80 (0.45)	4.26	0.04	0.61
	Self-help	3	5.67 (0.76)			
Self-help materials’ organization	Blended	5	7.00 (0.00)	6.56	0.01	0.94
	Self-help	3	4.67 (0.76)			

As reported in Table 3 the results regarding the personal evaluation of impact, specifically the perceived improvements due to the interventions, reveal higher mean scores for the blended and peer-to-peer groups compared to the self-help group. The subjective evaluation of interaction improvement also varied significantly between the groups, with greater improvement reported in the blended and peer-to-peer groups. Similar results were found for the increase in the number of relationships, with significantly higher scores indicating a greater increase in relationships in the blended and peer-to-peer groups. Although not statistically significant, some differences were also observed in the perceived increase in coping resources: higher scores were attributed to the peer-to-peer and blended groups compared to the self-help group. All these comparisons reported a medium-large effect size (ε^2^ ≥ 0.13).

**Table 3:** Group comparison in the personal evaluation of the effects of the intervention.

	Group	*N*	*M* (SD)	χ^2^	*p*-value	ε^2^
Interaction improvement	Blended	5	6.20 (0.45)	6.80	0.03	0.576
	Peer-to-peer	5	6.40 (0.89)			
	Self-help	3	4.33 (0.58)			
Number of relationships increased	Blended	5	6.60 (0.89)			
	Peer-to-peer	5	6.40 (0.89)	5.85	0.05	0.49
	Self-help	3	4.17 (1.26)			
Wellbeing improvement	Blended	5	6.00 (1.23)			
	Peer-to-peer	5	6.20 (1.10)	2.03	0.36	0.17
	Self-help	3	5.17 (0.29)			
Coping resources increased	Blended	5	6.00 (1.00)			
	Peer-to-peer	5	6.20 (0.84)	3.52	0.17	0.32
	Self-help	3	4.83 (0.76)			

Responses collected with the use of the open-ended questions were thematically analyzed and grouped in four thematic areas: motivations to participate in the intervention, strengths and weaknesses of the interventions, and suggestions to improve the intervention. A summary of themes per group with useful participants’ citations, are reported in Tables 4–6.

**Table 4:** Overview of the main themes identified in the blended group’s open answers.

Thematic areas	Themes	Participants’ citations
Motivations to participate in the intervention	The opportunity to meet new people	“I saw an opportunity to meet new people” (P5)
	Sharing experience and points of view	“I thought sharing my experiences and get to know others’ experiences would enlight me” (P3)
	Expectation of an interesting experience	“I thought it would be an interesting experience” (P4)
Strengths of the intervention	Meeting new people	“Meeting other international students” (P2)
	Comfortable and collaborative group atmosphere	“It was a really comfortable space without judgment” (P5)
	Relevant and stimulating topics	“Everyone had great ideas and stimulating input that was always very eye opening and enjoyable to hear” (P1)
	Sharing experiences	“Everyone could share something even the bad stuff” (P3)
	Shared experiences	“Everyone is going through the same things” (P5)
	Less loneliness	“I feel less lonely” (P4)
	New perspectives, ideas, and hobbies	“New perspectives on life… there are also many new hobbies and habits I’ve taken up as a result of these conversations” (P1)
	Relevant and stimulating topics	“Speak with peers about relevant topics in our lives” (P1)
	Safe group atmosphere	“I felt like it was a safe place to talk about my struggles” (P4)
Weaknesses of the intervention	Lack of participation	“More the people, better the understanding” (P5)
	Few meetings (would like to have more meetings)	“Maybe it could’ve been longer since it’s only once a week” (P4)
Practical suggestions to improve the intervention	More topics	“There were still some factors which were not discussed, in my opinion. So there is still room to add more topics” (P5)
	More meetings	“I would’ve loved to do it twice a week” (P4)
	Verify people availability to participate (participation)	“You would interview with the candidates to choose them” (P3)

**Table 5:** Overview of the main themes identified in the self-help group’s open answers.

Thematic areas	Themes	Participants’ citations
Motivations to participate in the intervention	To improve my mental health	“To see the shortcoming of myself and to improve them” (P1)
Strengths of the intervention	Practical and helpful materials	“It is short, comfortable, helpful and practical” (P2)
	Working and focusing on myself for wellbeing	“I can pursue well-being by myself and with some techniques” (P2)
	Social support gives hope	“To see group of people together gives people hope” (P1)
	Relevant and good-organized topics	“Every suggestion is clear and very useful” (P2)
Weaknesses of the intervention	Online modality: no face-to-face interaction	“It is important to have it face to face for everyone” (P1)
Practical suggestions to improve the intervention	Face to face sessions	“Arrange face-to-face lessons” (P2)
	More suggestions	“More personal suggestions and support” (P3)

**Table 6:** Overview of the main themes identified in the peer-to-peer-group’s open answers.

Thematic areas	Themes	Participants’ citations
Motivations to participate in the intervention	Meeting new people and connecting	“Main reason is that I want to meet new international people” (P1)
	Sharing challenges and experiences of international students	“Finding a Place to share similar experiences” (P5)
Strengths of the intervention	Cultural and thoughts exchange	“Exchanging different cultures and thoughts” (P1)
	Commonality and team working	“Working together in all session” (P4)
	Learning about challenges of international students	“Learnt something about the students challenges” (P2)
	Better communication	“Lose the fear of talking about my bad and good experience” (P5)
	Meeting new people	“People or new friends will be more suitable words” (P4)
	Interesting topics and activity organization	“Interesting subjects for talking every” (P3)
	Engagement	“Engagement” (P1)
Weaknesses of the intervention	Specific topics	“Some parts were boring but just a little” (P3)
	Chosen period	“May be the chosen period for this activity” (P4)
	Lack of participation	“Not participating all member that end up talking with 2 or 3 persons” (P5)
Practical suggestions to improve the intervention	Greater participation and more people	“Reach to more people so more people can connect” (P1)
	Creative and applicative games	“And more interesting games to play together no just talking” (P3)
	Period (not exam session)	“It would be good if this event starts in the beginning of the semester” (P4)

As reported in Table 4, participants in the blended group chose to attend the group activities primarily to meet new people and have a space and opportunity to compare themselves with peers. The blended activities resulted in the lessening of feelings of loneliness, and in the elaboration of new perspectives and points of view by sharing reflections and experiences with others in the group, and elaborating them alone with the use of the online self-help materials.

The participants expressed the desire for the blended group to last longer and involve more participants in order to enrich the possibilities of getting to know different points of view and experiences.

As reported in Table 5, those participants who received only the online self-help materials, chose this intervention to improve their mental health. Thanks to the online self-help materials, the participants reported to have focused more on their wellbeing and recognized the importance of social support. Participants enjoyed the topics dealt with by the online self-help materials, and found them relevant and consistent with their everyday life challenges, even though they lamented the absence of face-to-face connection with peers, indicating that this would have enriched the experience itself.

As reported in Table 6, participants who took part in the peer-to-peer group reported having chosen this activity to meet new people and have the possibility to share their experiences and difficulties as international students with peers. The participants enjoyed the group activities and the exchange of ideas and experiences that they experienced as part of the group, underlining that this sharing of ideas and experiences enriched them, as well as provided them with better communication strategies and new relationships with peers. The participants lamented the poor participation in the group sessions and the difficulties attending the meetings due to the academic exams period.

### 3.2 The impact of the intervention and differences between groups

**Figure 2:**
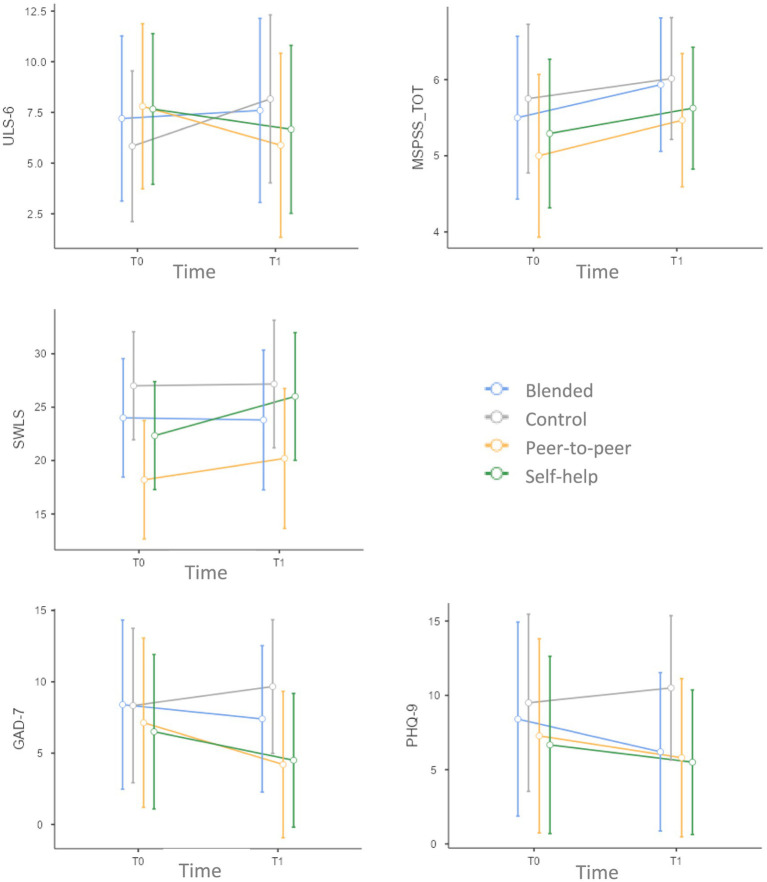
Comparisons between groups on the variables measured through the questionnaires.

The comparison within each group between the scores of the self-report questionnaires at the baseline (T0) and post-intervention (T1) showed that ULS-6 scores were higher for the control group and lower for the peer-to-peer group at T1 compared to T0. At T1, the GAD-7 and PHQ-9 scores increased and exceeded the clinical threshold only in the case of the control group. In the blended, peer-to-peer and self-help groups, lower scores were observed for the GAD-7 and the PHQ-9 test at T1 compared to T0. At T1, all the groups, except the control group, exhibited higher life satisfaction scores compared to T0. These differences between groups can be observed in Figure 2 and are confirmed by the medium-high effect size (|*ρ*| ≥ 0.30) associated to these comparisons even if paired Wilcoxon tests were not significant (see Table 7).

**Table 7:** Comparisons of the questionnaire scores at baseline (T0) and at post-intervention (T1) for the each group.

			Baseline T0	Post-intervention T1			
	Group	*N*	*M* (SD)	*M* (SD)	*W*	*p*	*ρ*
ULS-6	Blended	5	7.20 (4.97)	7.60 (4.98)	4.50	1.00	−0.10
	Control	6	5.83 (4.49)	8.17 (5.46)	1.50	0.14	−0.80
	Peer-to-peer	5	7.80 (4.44)	5.88 (4.55)	12.00	0.31	0.60
	Self-help	6	7.67 (3.45)	6.67 (4.23)	9.50	0.68	0.27
MSPSS	Blended	5	5.50 (0.96)	5.93 (0.78)	0.00	0.10	−1.00
	Control	6	5.75 (0.89)	6.01 (0.48)	8.00	0.67	−0.24
	Peer-to-peer	5	5.00 (1.31)	5.47 (0.90)	3.50	0.34	−0.53
	Self-help	6	5.29 (1.32)	5.63 (1.33)	6.00	0.44	−0.43
GAD-7	Blended	5	8.40 (5.13)	7.40 (6.03)	11.50	0.34	0.53
	Control	6	8.33 (7.63)	9.67 (7.87)	1.00	0.42	−0.67
	Peer-to-peer	5	7.13 (7.11)	4.20 (2.17)	11.50	0.34	0.53
	Self-help	6	6.50 (4.85)	4.50 (3.56)	16.00	0.29	0.52
PHQ-9	Blended	5	8.40 (5.51)	6.20 (2.28)	12.00	0.28	0.60
	Control	6	9.50 (9.07)	10.50 (9.05)	2.50	0.46	−0.67
	Peer-to-peer	5	7.28 (6.85)	5.80 (4.44)	7.50	0.46	0.50
	Self-help	6	6.67 (5.47)	5.50 (3.73)	15.00	0.39	0.43
SWL	Blended	5	24.00 (6.04)	23.80 (3.11)	5.00	0.58	−0.33
	Control	6	27.00 (5.55)	27.17 (6.24)	7.50	1.00	0.00
	Peer-to-peer	5	18.20 (5.26)	20.20 (9.52)	4.50	1.00	−0.10
	Self-help	6	22.33 (6.56)	26.00 (7.46)	2.50	0.22	−0.67

Given the high number of people who did not complete the study, the researchers compared their scores on the standardized questionnaires at the baseline with the scores of the sample that completed the study. The results of this comparison are reported in Table 8. Significant differences were found between the groups in the ULS-6, GAD-7, and PHQ-9 questionnaires. The non-completers’ group reported significantly higher levels of perceived loneliness, anxiety and depression. All these comparisons had a moderate-large effect size (|ρ| ≥ 0.30).

**Table 8:** Comparison between the non-completers’ group (*N* = 27) and the completers’ group (*N* = 22).

	Non-completers’ group *M* (SD)	Completers’ group *M* (SD)	*U*	*p*	*ρ*
ULS-6	10.26 (4.30)	7.09 (4.09)	176.00	0.01	0.41
MSPSS_TOT	5.03 (1.04)	5.40 (1.09)	240.00	0.26	0.19
GAD-7	11.26 (5.45)	7.58 (5.90)	187.50	0.03	0.37
PHQ-9	11.07 (6.00)	7.97 (6.54)	197.50	0.05	0.36
SWL	20.30 (6.82)	23.05 (6.34)	243.00	0.28	0.18

## 4 Discussion

The present study aimed to test the methods and procedures of an intervention to reduce social isolation and loneliness in international students, with the primary objective of evaluating its feasibility and relevance for participants, exploring potential effects and experiences that warrant further investigation in a subsequent, more comprehensive study (Thabane et al., 2010). The intervention conditions were specifically developed to meet international students’ needs and to allow them to face the challenges of living in a foreign country. Activities and materials were developed based on the conceptual framework of the PCP (Kelly, 1955) and by considering social support as a fundamental construct for well-being, especially in terms of meaningful relationships (Lee and Goldstein, 2016; Umberson and Karas Montez, 2010; Ryff, 1989).

The pilot study proposed three different types of intervention (blended, online self-help or peer-to-peer) encompassing group activities and self-help materials. The qualitative results show that the interventions including in-presence group activities provided a social context in which the participants could experience social connectedness, and enter in relationship with peers, while the online self-help intervention left the reflection process to the individuals and their environment, limiting the potential for experiencing social connections.

The comparison of the blended, self-help, and peer-to-peer interventions with the control condition allowed for an assessment of the feasibility and effectiveness of each intervention modality in addressing social isolation and loneliness among international students, and in fostering their well-being. The participants’ satisfaction with the interventions, and the individual relevance of the content and activities, are essential for assessing their potential impact and feasibility for larger-scale implementation (Thabane et al., 2010).

The findings reveal that the interventions that included in-person group activities (i.e., the blended and peer-to-peer interventions) were perceived as more relevant and more functionally organized compared to the intervention that only involved online self-help materials. Furthermore, the participants reported that the topics proposed and discussed in the blended and peer-to-peer interventions, which included group activities, were relevant to their lives, and encouraged stimulating reflections on their experiences, leading to the development of new perspectives.

On the other hand, the participants in the self-help group condition reported to have found the online materials useful and relevant when it came to addressing many challenges and fostering their well-being (Schotanus-Dijkstra et al., 2015; Walsh et al., 2018), but complained about the absence of face-to-face interaction and suggested integrating the intervention with in-person sessions, while acknowledging the significance of offline relationships in strengthening well-being and social support (Nowland et al., 2018).

Notably, in terms of satisfaction with the interventions, the results show that the blended group evaluated the self-help materials as being relevant, satisfactory, and organized in a more effective way compared to the self-help group. These findings emphasize the necessity of transforming self-help materials’ insights and feedback into active and direct experiences, thereby increasing the materials’ relevance and increasing the beneficial impact on well-being. This observation is consistent with Esposito and colleagues’ findings (Esposito et al., 2017), who claimed that reflexive processes proposed by self-help materials have only limited effects when exclusively used for individual thinking, but they can have greater potential when involving a process of shared meaning co-construction among participants in a collective context.

Comparing the blended and peer-to-peer groups, no significant differences are noted in terms of satisfaction, relevance, improvement in connection and well-being, a result that is possibly due to the same structure of activities and contents being involved. Concerning the peer-to-peer group, open-ended responses focused on the possibility of new social connections, which could enhance feelings of self-acceptance and social support, positively impacting on depression, anxiety, and feelings of helplessness (Bessaha et al., 2020; Kotwal et al., 2021). Blended group participants’ responses however, stressed the relevance of the non-judgmental atmosphere of the group, an element that supported them in discussing significant topics and sharing personal experiences.

The blended and peer-to-peer intervention facilitated the free exchange of experiences and ideas in a secure and cooperative environment, leading to the establishment of new social connections rooted in the common experience of being international students. Uniting individuals with shared experiences, indeed, reduces feelings of loneliness and validates individual constructs (Bessaha et al., 2020; Carr et al., 2003; Yakunina et al., 2011). Moreover, working in groups offered participants a chance to reshape their meaning systems and construe novel perspectives, helping them to enrich their constructs or elaborate new anticipations and resources. The present findings support Garcia-Martínez et al.’s (2021) hypothesis that report how the process of construing shared meanings can enhance communication and foster transformative interactions.

The statistical analyses run to verify the impact of each intervention on the standardized questionnaires did not reveal significant results. Nevertheless, the effect size was large enough to suggest an increase of perceived social support and wellbeing and a decrease of anxiety, depression in the blended group and an increase of perceived social support and a decrease of anxiety, depression and loneliness in the peer-to-peer group. On the contrary, the control group reported an increase of anxiety, depression and loneliness and did not report any effects of change on social support and wellbeing. The group that only used self-help material reported an increase of perceived social support and well-being, but also an increase of anxiety and depression.

Only perceived social support increased significantly over time across all groups, including the control group, but there was no evidence to attribute such effect to the type of intervention. A hypothesis of interpretation could be found in the evolutionary function of loneliness (Cacioppo et al., 2014), suggesting that loneliness can motivate reconnection with others, leading to improved perceived social support over time without the need for specific interventions (Masi et al., 2011). Other hypotheses might be linked to the specific context of the study: first, being involved in the study might in itself have made participants feel more supported than before the participation in the study, and second, the period when the study was conducted (2022–2023) was a moment of transition following the restoration of social and in-person academic activities post-COVID19 pandemic when students perceived high social support (Bersia et al., 2024).

Another potential explanation of the results obtained in the measure of perceived social support, but also in the loneliness questionnaire, regards the nature of interventions to address loneliness, which are typically focused and designed to increase social connections and reduce social isolation, rather than specifically focusing on reducing experiences of loneliness (Wigfield et al., 2022). The results of the present study suggest that the proposed interventions contributed to increase the perception of social support whereas only the peer-to-peer intervention contributed to decrease the feelings of loneliness but not to increase perceived social support. This is an interesting data that deserves to be further explored and that suggests that peer-to-peer intervention might be the modality of choice to address loneliness.

The lack of statistical significance in the other questionnaire scores raises questions about the reasons behind this outcome if compared to the subjectively reported change in participants’ wellbeing and social connectedness, and to the differences observed in the descriptive analyses, also confirmed by the effect size. Hypothetical reasons that have been acknowledged and discussed within the research team include the small sample size and the high non-completers’ rate, which may have contributed to the lack of statistical significance. Furthermore, the PCP theoretical framework (Kelly, 1955) based on which the interventions were designed, focuses on the exploration and elaboration of participants’ personal experiences and meanings, and, by its nature, the framework avoids standardization. This may have led to difficulties in using standardized questionnaires to measure such experiences, as they may oversimplify complex experiences. In this sense, the PCP framework may have constrained the capacity of the administered tests to measure intervention-induced changes. This limitation could arise from the standardized nature of the tests: the questionnaires used could not assess changes in the personal processing and elaboration of the constructs they analyze, which constituted a pivotal focus of the intervention.

Moreover, the language of administration of the tests and their cultural sensitivity may have played a crucial role. All of the standardized tools employed in fact were validated involving Anglo-American populations, while the pilot study participants who completed these measures, were mainly non-native English speakers and non-Anglo-American. Such cultural specificity, and potential difficulties in understanding and correctly interpreting the questions provided in a vehicular language, could have resulted in the tests’ lack of sensitivity to the respondents’ cultural diversity and language ability, compromising the results of the tests. Thus, while the collaborative interactions among students could have helped them overcome communication barriers during the group activities, potential language barriers may have influenced the results and compromised the assessments. These results raise different questions regarding the consistency and feasibility of the standardized instruments adopted, and the possibility of considering alternative measures for future evaluations.

Moreover, due to the close timing of the questionnaires’ administration (within approximately 2 months), the recorded scores could have been influenced by the test–retest effect and memory bias, potentially underestimating the actual change. Another important element to consider is that the majority of the participants in the present study were enrolled in psychology degree programs, potentially influencing the results and scores attributed due to familiarity bias. In fact, familiarity with professional psychological interventions and psychological tests may have influenced their completion and the evaluation of the intervention impact itself.

As a matter of fact, participants both in the blended and peer-to-peer groups, reported a willingness for longer face-to-face group activities, to help them develop solid relationships and build satisfactory social support networks that could positively impact their psychosocial wellbeing. The study was designed to encompass 8 sessions based on the existing literature (Kotwal et al., 2021; Osborn et al., 2021), which frequently suggests a duration of 6–8 sessions as optimal for implementing these intervention modalities. Despite this, as reported by the participants, and according to the literature (Brunelli et al., 2016), a longer duration could enhance the development of meaningful relationships and impact on clinically-relevant dimensions of mental health. This suggestion should be taken into account for the future implementations of interventions.

Furthermore, data show that in the blended group all participants, with one exception, had previous experiences with psychological support, whereas no one in the peer-to-peer group, and only one in the self-help group, reported previous experiences with psychological support. Taking those elements into consideration, it is plausible to hypothesize that the outcomes observed in the blended group might underestimate the potential benefits of the blended intervention. Our study suggests that previous psychological support experience could have confounded the results, potentially diminishing the specific impact of the intervention: previous interactions with mental health professionals may have already enriched participants’ psychological and social resources, which are the same resources the interventions aimed to provide. Interestingly, the blended group had a greater number of participants with prior experience of psychological support, suggesting that individuals accustomed to a specific intervention structure may have been inclined toward seeking professional-led intervention rather that peer-to-peer activities. Additionally, when psychological support is ongoing, measures of mental health and well-being might be more critical, carrying the risk of subsequently influencing the results. Future investigation, taking into account those elements, is necessary to elaborate further our results and hypothesis.

Another relevant element to be discussed relates to the high non-completers’ rate between baseline and post-intervention assessment. Comparative analyses between the non-completers’ group and the group that completed the interventions were carried out to investigate elements that could explain potential causes. Those analyses revealed significantly higher levels of loneliness, anxiety and depression raising questions about whether participants with elevated levels of anxiety and loneliness might perceive the group intervention as overly threatening, or the self-help materials as irrelevant for their needs. These findings may indicate that the treatment completers may not adequately represent the general population, while suggesting that administering a preliminary clinical screening before the start of the intervention may be pivotal for future research. Such screening could help orient different participants to more suitable modalities of intervention based on personal characteristics and psychological resources, hopefully preventing the high non-completers’ rate. Moreover, more effective recruitment strategies might be implemented such as organizing a pre-intervention meeting or call to present the aim and modalities of the intervention and allow participants to better understand the study and choose if they wanted to take part in it. Another option might be to involve the students in the recruitment process through the organizations of social initiatives where the students might participate and feel committed, thus lowering the threat of participating in an intervention promoting social connection and giving them an active role in organizing and planning the intervention also according to their needs and constrains (e.g., choosing a period of the year that is more suitable for them).

Even though not as expected, the results collected in this pilot study support the possibility that tailored interventions improve social connectedness and wellbeing, underlining the importance of providing spaces to meet other people and share relevant experiences, while dealing with specific challenges and disruptions. The present pilot study in this sense provides initial data and considerations which should be taken into account when designing and implementing future versions of tailored interventions to support international students in adapting to the new situation, fostering their social connectedness with peers and increasing their wellbeing. The results provide interesting starting points that foster the possibility of reflecting on and discussing the importance of creating and implementing tailored interventions, involving multidisciplinary activities and resources, such as the digital and group ones, responding to the actual needs of these populations.

### 4.1 Limitations and future directions

The present study reported a number of limitations. First, the small sample size. In fact, the power analysis conducted during the research design had originally hypothesized a sample size of 48 participants to achieve a statistical power of 0.80 with a medium effect size (*d* = 0.25) in a mixed ANOVA analysis, when the significance level was set at *α* = 0.05. However, due to the high non-completers’ rate (55.10%), and the limitations in the recruitment of the participants, the initial number of participants (49) was significantly reduced, resulting in a final sample size of 22. The high non-completers’ rate is one of the study’s key limitations, and may have altered the significance of the findings and limited the strength of the conclusions reached from this study (Bouwman et al., 2017). According to the literature, interventions performed in less formal settings and with more mobile populations, as well as online self-help programs, have greater non-completer rates (Masi et al., 2011; Osborn et al., 2021). Looking to future implementations of the present intervention study, it is fundamental to recruit an additional 25% of participants over the number found through the power analysis, in order to mitigate the non-completers’ effect.

Second, the study was conceived as a RCT, but due to the limited sample size and challenges in recruiting participants, the strict structure of a randomized controlled trial was abandoned, acknowledging the potential weaknesses inherent in non-randomized studies for psychological research. Therefore, the study consists of a group comparison study, which can present important design flaws, including selection bias. In fact, when people are not randomly assigned to intervention or control groups, those participating in the intervention conditions may vary from people in the control condition in ways that might have an impact on the study’ findings, underlining the importance of reducing such bias with RCT studies (Masi et al., 2011) for future implementations of our research.

Following the implementation of a non-randomized research design, it is possible to observe a different composition of the groups assigned to the various proposed conditions: while the number of participants in each group is approximately homogeneous when non-completers are excluded, it is not equally homogeneous in terms of gender, degree program, and familiarity with psychological support. Looking at the overall sample, females are predominant in the blended, self-help, and control groups, with the peer-to-peer group being the only one with a majority of male participants.

The literature suggests that female international students tend to have higher levels of social support than their male counterparts, and are more likely to seek help from others in managing higher levels of social and cultural distress (Alsubaie et al., 2019; Dwyer and Cummings, 2001; Mahanta and Aggarwal, 2013). This might be the reason for the higher percentage of female participants in the interventions proposed, leading to non-homogeneous group composition. In this sense, gender may have influenced the participants’ responses to loneliness experiences, but it is challenging to establish how this differing composition may have impacted the results.

Another variable to consider is that the study included international students enrolled at the University of Padova, on various degree programs. However, the PhD category has unique challenges such as navigating a demanding academic environment, grappling with a wavering sense of self-worth, apprehensions of isolation, diminished motivation, self-doubt regarding intelligence, and feelings of inadequacy (Son and Park, 2014), experiences that might benefit more from tailored and targeted interventions. Despite the low number of PhD students, the self-help group is primarily composed of them, indicating potential differences in intervention outcomes due to the distinct characteristics of this student population. The PhD student population should be considered as a separate group in the future implementation of the interventions.

## 5 Conclusion

In conclusion, this study provides valuable insights into interventions targeting social isolation and loneliness. The results emphasize the significance of group-based approaches in enhancing personal well-being and perceptions of social support. However, achieving statistical significance in self-report standardized questionnaire analyses was inconsistent, underscoring the need for further investigation.

The study highlights the importance of face-to-face group interactions in fostering a sense of belonging and connectedness among participants, as well as ways to construct and co-construct one’s personal meaning. Despite limitations such as a high non-completer’ rate and nonhomogeneous group distribution, the feasibility and relevance reported by participants suggest a beneficial impact of the group in-person interventions on interaction improvement and resource development. Future research should address these limitations by considering larger participant samples and implementing longitudinal designs to capture the long-term effects of those interventions, allowing for a nuanced exploration of their impact on well-being and social support over time. Finally, randomized controlled conditions could enhance study rigor and enrich findings.

## Data Availability

The datasets presented in this study can be found in online repositories. The names of the repository/repositories and accession number(s) can be found at: Data are available on OSF at the link https://osf.io/zypjv/.
